# Smaller CO_2_ injection volume and lower gastric pressure induce bothersome symptoms in drug-resistant functional dyspepsia patients with less frequent belching

**DOI:** 10.1371/journal.pone.0271456

**Published:** 2022-07-14

**Authors:** Eri Momma, Saori Kanai, Yoshimasa Hoshikawa, Mai Koeda, Tomohide Tanabe, Shintaro Hoshino, Noriyuki Kawami, Mitsuru Kaise, Katsuhiko Iwakiri

**Affiliations:** 1 Department of Gastroenterology, Nippon Medical School, Graduate School of Medicine, Bunkyo-ku, Tokyo, Japan; 2 Endoscopic Center, Nippon Medical School Hospital, Bunkyo-ku, Tokyo, Japan; University of Malaya Faculty of Medicine, MALAYSIA

## Abstract

**Background:**

The relationship between bothersome symptoms and gastric pressure or CO_2_ injection volumes in drug-resistant functional dyspepsia (FD) patients remains unknown; therefore, this relationship was examined in drug-resistant FD and non-FD patients.

**Methods:**

Thirty drug-resistant FD patients and 30 non-FD patients were recruited. Gastric pressure was assessed using an external pressure transducer, and the CO_2_ injection volume was measured using an endoscopic CO_2_-supplied device and flow meter. The following variables were examined: gastric pressure at baseline and gastric pressure and the CO_2_ injection volume when patients initially felt abdominal tension and bothersome symptoms following the CO_2_ injection. The relationship between belching and bothersome symptoms was also investigated.

**Results:**

No significant differences were observed in basal gastric pressure between the groups. Initial and bothersome symptoms in the upper abdomen in drug-resistant FD patients developed at a significantly lower gastric pressure and significantly smaller CO_2_ injection volume than in non-FD patients. The frequency of belching was significantly lower in the drug-resistant FD group than in the non-FD group.

**Conclusion:**

Bothersome symptoms in drug-resistant FD patients develop at a lower gastric pressure and smaller CO_2_ injection volume than in non-FD patients. These patients also had difficulties with belching.

## Introduction

Although there is a lack of organic disease in the upper gastrointestinal (GI) tract, patients with functional dyspepsia (FD) have upper abdominal symptoms and their quality of life (QOL) is markedly reduced [1–3]. Primary causes of FD include gastric motility dysfunction and gastric hypersensitivity [4–7]. However, there is currently no routine method for evaluating gastric motility dysfunction or gastric hypersensitivity. In our previous study, we developed a method to measure gastric pressure and the CO_2_ injection volume while injecting CO_2_ during endoscopy, and demonstrated that, initial upper abdominal symptoms after CO_2_ injection develop with significantly lower gastric pressure and CO_2_ volume in drug-resistant FD patients who are strongly suspected of having gastric hypersensitivity [8], compared with non-FD patients. We also reported that the sensitivity and specificity to drug-resistant FD were 81.5% and 81.5%, respectively, with a cut-off gastric pressure of 12.7 mmHg. Similarly, with a cut-off CO_2_ injection volume of 1.25 L, the sensitivity and specificity of CO_2_ volume to resistant FD were 85.0% and 96.3%, respectively. Thus, the measurement of continuous gastric pressure and CO2 injection volume may be an effective measure to evaluate gastric mucosal hypersensitivity. However, little is known about the relationship between bothersome symptoms and gastric pressure or CO_2_ injection volume in drug-resistant FD patients. Then, in this study, we investigated the relationship between them using the previously described our method in drug-resistant FD patients and non-FD patients. We also investigated the association between belching and bothersome symptoms.

## Methods

Drug-resistant FD patients who exhibited dyspepsia symptoms for longer than 6 months and non-FD patients without GERD symptoms, such as heartburn and regurgitation, were recruited for this case-control study at a single center (Department of Gastroenterology, Nippon Medical School Hospital) between March 2021 and October 2021. All subjects were confirmed to have no organic diseases by endoscopy. Data on the drug-resistant FD and non-FD patients were collected during endoscopy.

Drug-resistant FD is defined as the presence of at least 1 symptom of dyspepsia other than “belching” on the revised F scale [9] with a score of 4 points (frequency of symptoms: always) or at least 1 symptom of dyspepsia other than “belching” with a score >3 points (frequency of symptoms: often) and a total dyspepsia symptom score >8 points [8]. Non-FD is defined as a dyspepsia score ≤1 (frequency of symptoms: occasionally) and a total dyspepsia symptom score, excluding “belching”, ≤3 points [8].

Patients with the absence of systemic or metabolic disease, negative results in the fecal occult blood test (the 2-day method), and normal abdominal ultrasonography were classified as the drug-resistant FD group. The symptoms of patients in this group were not attenuated by the administration of proton pump inhibitors (PPIs) or prokinetic agents. In the present study, FD was diagnosed according to the criteria of evidence-based clinical guidelines for FD by the Japanese Society of Gastroenterology [10].

Non-FD patients included those who had gastric and/or duodenal ulcer scarring, including after *Helicobacter pylori* eradication, those who were in a follow-up period for chronic gastritis or atrophic gastritis and those who underwent endoscopy for the purpose of screening for upper GI tract. To investigate the association between belching and bothersome abdominal symptoms, patients who were suspected of having GERD in previous endoscopy or medical interviews were excluded from the non-FD group because belching may be associated with GERD.

Endoscopic examination was carried out by the same endoscopist (EM or KI) while conscious. The patients were on medications at the time of examination as we did not observe any effects of the medications on symptoms.

**Fig 1:**
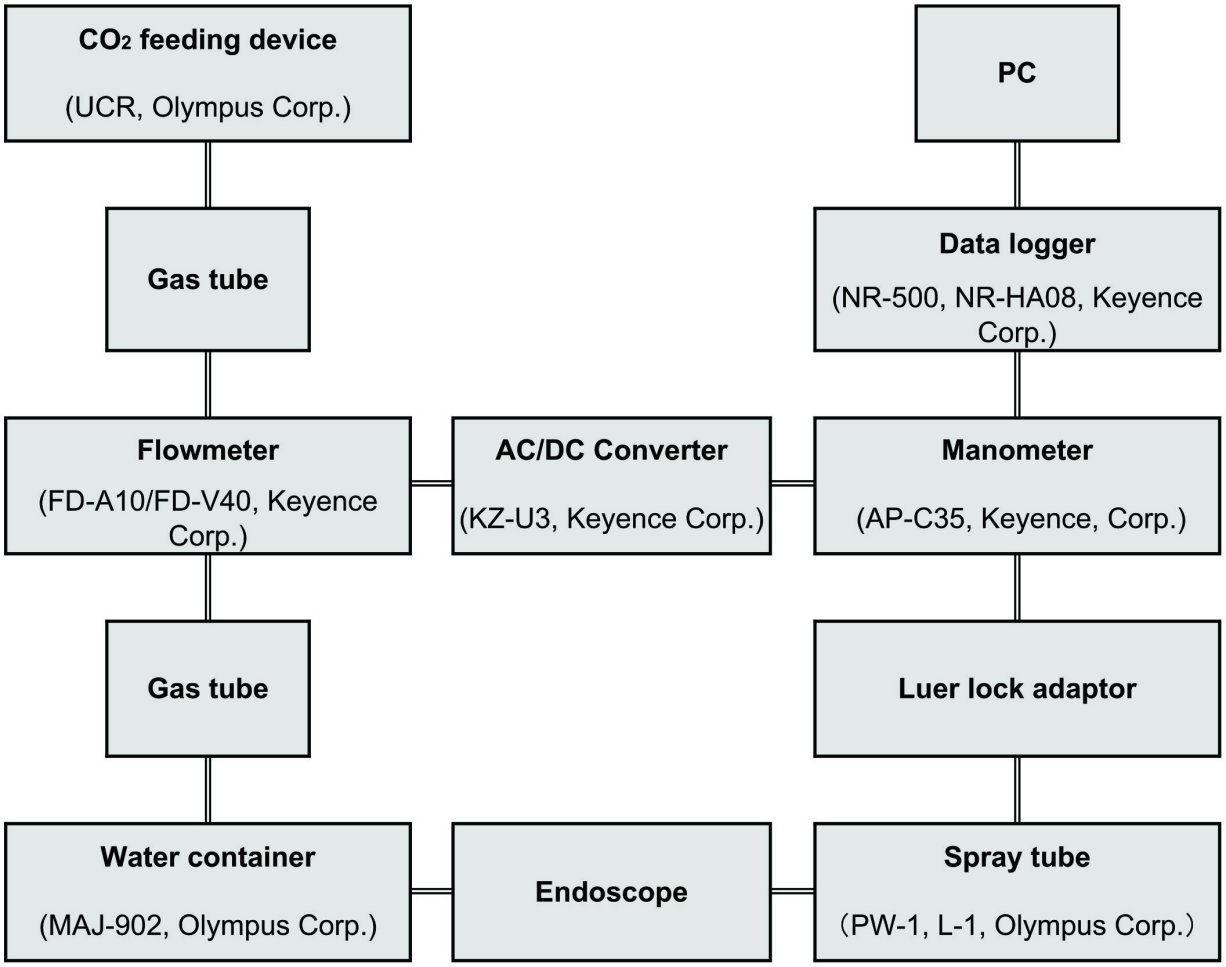
Outline of gastric pressure and CO_2_ injection volume measurements.

An endoscope (H290, Tokyo, Olympus Corp.) was inserted through the oral cavity and its tip was positioned inside the fornix. The spray tube was moved through the forceps hole and its tip was fixed slightly past the endoscope. Any gastric juice or clogs in the spray tube were removed by flushing with a small amount of air, and this was followed by the measurement of basal gastric pressure. If the amount of liquid in the stomach was large, aspiration was conducted to remove a small amount and create an empty space for the measurement of basal gastric pressure. Breathing was shallow and basal gastric pressure was assessed as the intermediate pressure of breathing in a stable state. The air supply button was repeatedly pressed to deliver a continuous injection of CO_2_ at a constant speed, and gastric pressure and the CO_2_ injection volume were assessed [8]. Gastric pressure and CO_2_ injection volume measurements are outlined in Fig 1. The tip of the spray tube was connected to an external pressure sensor (AP-C35, Osaka, Keyence Corp.) for continuous measurements of gastric pressure.

An endoscopic monitor displayed gastric pressure data, which were stored on a PC by a data collection system (NR-500, NR-HA08, Osaka, Keyence Corp). The volume of CO_2_ being injected was measured continuously by a flow sensor and meter (FD-A10/FD-V40A, Osaka, Keyence Corp), which were installed between the CO_2_ insertion device (UCR, Tokyo, Olympus Corp.) and the endoscope, and this information was also shown on the monitor. Data were stored on a PC. Instructions were given to patients to indicate any sensation of upper abdominal tension during the CO_2_ injection by lifting their right hand, at which point gastric pressure and the volume of CO_2_ injected were recorded [8].

After injecting CO_2_ again, patients were instructed to raise their right hand when they experienced bothersome symptoms, and the gastric pressure and CO_2_ injection volume at that time were recorded. CO_2_ injection was terminated if they experienced “belching” after the onset of initial symptoms. The presence of belching before or at the time of the onset of bothersome symptoms was investigated.

Body mass index (BMI), the degree of gastric mucosal atrophy, and the presence of hiatal hernia were examined on the day of endoscopy. The Kimura-Takemoto Classification was employed to assess gastric mucosa atrophy [11]: the absence of atrophy was classified as C1 and its presence as C2-O3. A diagnosis of hiatal hernia was made when the length between the hiatus and lower margin of the esophageal palisade vessels was >2 cm, and lengths were separated into <2 cm and >2 cm. This study was conducted after approval (B-2021-456) by the ethics committee of Nippon Medical School. Written consent was obtained from each patient.

Calculations of the sample size were based on the estimated proportion of drug-resistant FD and non-FD patients with gastric hypersensitivity. Since patient backgrounds were similar in the present and previous studies, the same settings were used to calculate sample sizes as those described in the previous study. Accordingly, 56% of drug-resistant FD patients and 18% of non-FD patients were assumed to gastric hypersensitivity. Therefore, each group needed to include 30 patients in order to detect differences in at least 38% between groups by Fisher’s exact test with a two-sided alpha error of 0.05 and power of 0.80.

### Statistical analysis

Data are presented as medians (25–75 percentiles). The Mann-Whitney U test was used to compare the differences in age, BMI, gastric pressure and CO_2_ injection volume between groups. Fischer’s exact test was performed to compare sex, the presence of gastric mucosal atrophy, hiatal hernia and the presence of belching before or at the time when bothersome symptoms occurred between the groups. P<0.05 was regarded as significant.

## Results

### Clinical characteristics

The clinical characteristics of patients in each group are shown in the Table 1. Compared with the non-FD group, patients in the drug-resistant FD group were significantly younger. Furthermore, the proportion of patients with gastric mucosal atrophy was significantly lower, and their BMI was significantly lower than that of patients in the non-FD group. There was no significant difference in the proportion of patients with hiatal hernia and sex.

**Table 1:** Clinical characteristics and demographic data of drug-resistant functional dyspepsia (FD) and non-FD groups.

	Drug-resistant FD	non-FD	P value
Age	58.5 (48.0–70.0)	70.5 (66.0–74.0)	0.0049*
Sex (Male/Female)	12/18	15/15	0.6042**
Body mass index	20.0 (18.7–23.0)	23.8 (20.9–25.6)	0.0038*
Gastric atrophy (+/-)	3/27	21/9	<0.0001**
Hiatus hernia (+/-)	11/19	4/26	0.2516**

Statistical analysis by Mann-Whitney U test * or Fisher’s exact test**

### Basal gastric pressure

There was no significant (p = 0.0993) difference in basal gastric pressure between the groups (resistant group: 6.0 mmHg (4.5–7.3), non-FD group: 6.5 (5.8–7.5)).

### Gastric pressure and CO_2_ injection volume at the time of initial awareness of a feeling of tension in the upper abdomen

**Fig 2:**
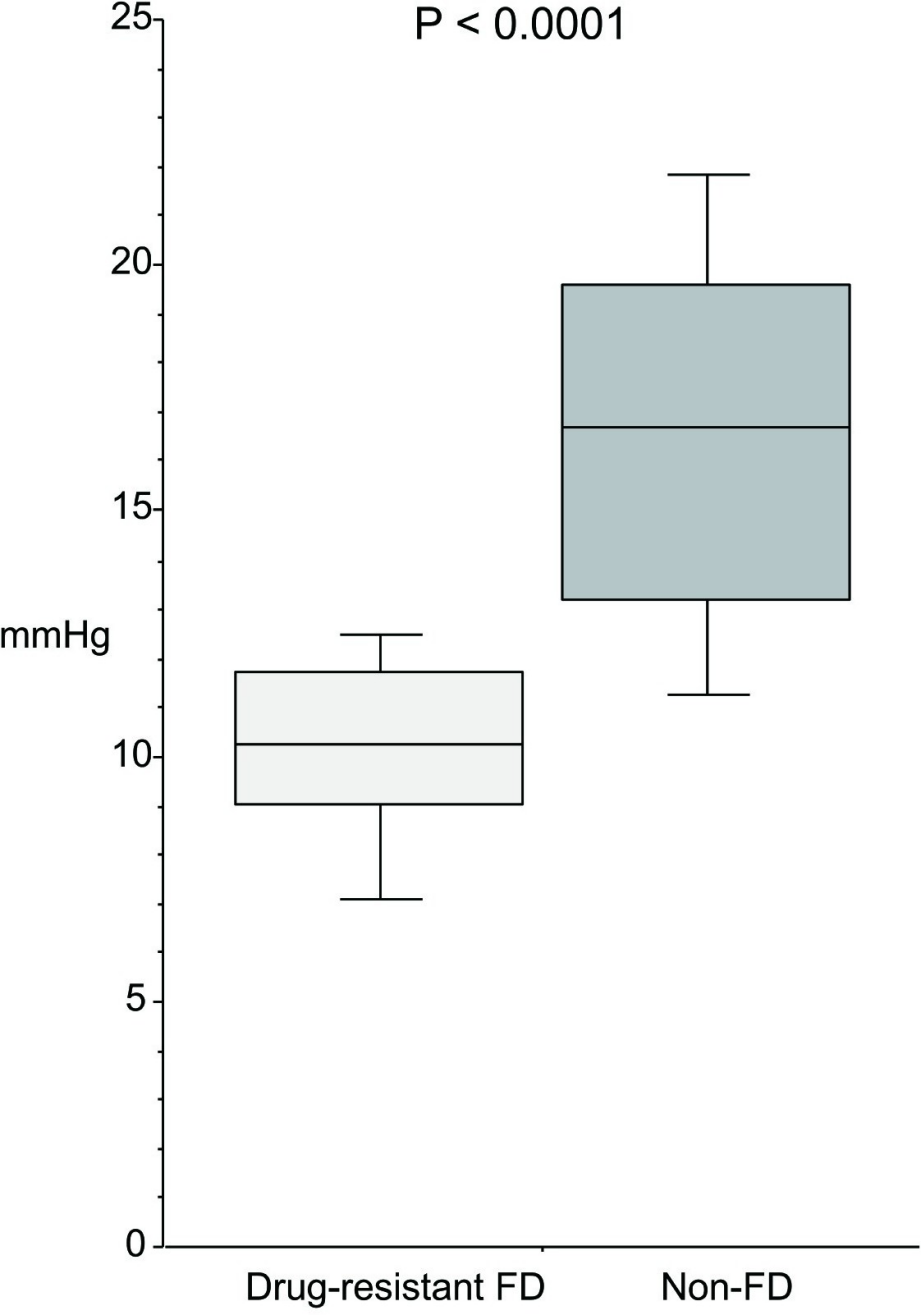
Gastric pressure at the time of awareness of a feeling of tension in the upper abdomen during a continuous CO_2_ injection.

**Fig 3:**
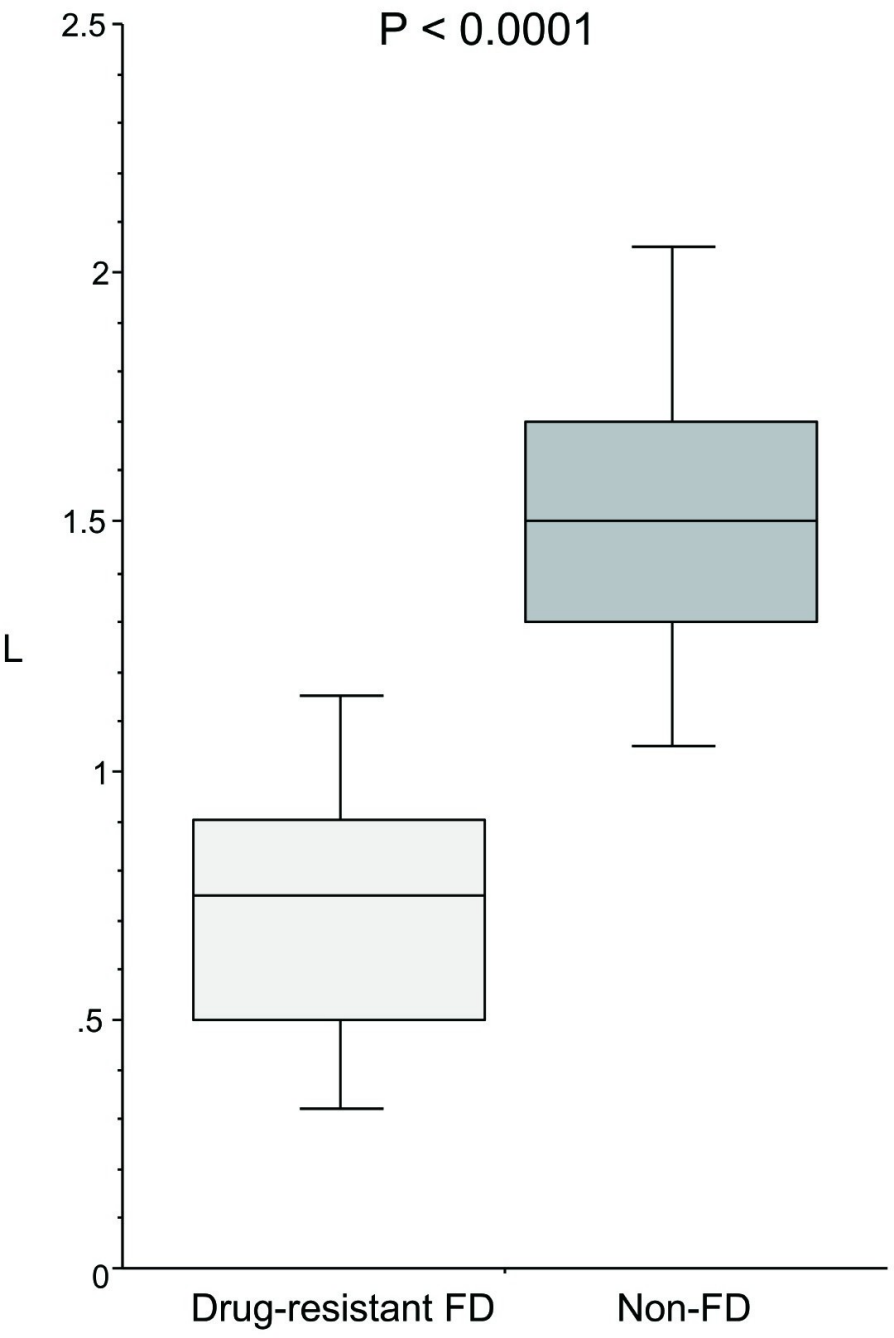
CO_2_ injection volume at the time of awareness of a feeling of tension in the upper abdomen during continuous CO_2_ injection.

Gastric pressure (Fig 2) at the time of initial awareness of a feeling of tension was significantly (p < 0.0001) lower in the drug-resistant FD group (10.3 mmHg (9.0–11.7)) than in the non-FD group (16.7 (13.2–19.6)). Similarly, CO_2_ injection volume (Fig 3) was significantly (p < 0.0001) lower in the drug-resistant FD group (0.8L (0.5–0.9)) than in the non-FD group (1.5 (1.3–1.7)).

### Gastric pressure and CO_2_ volume at the time of awareness of bothersome symptoms in the upper abdomen

**Fig 4:**
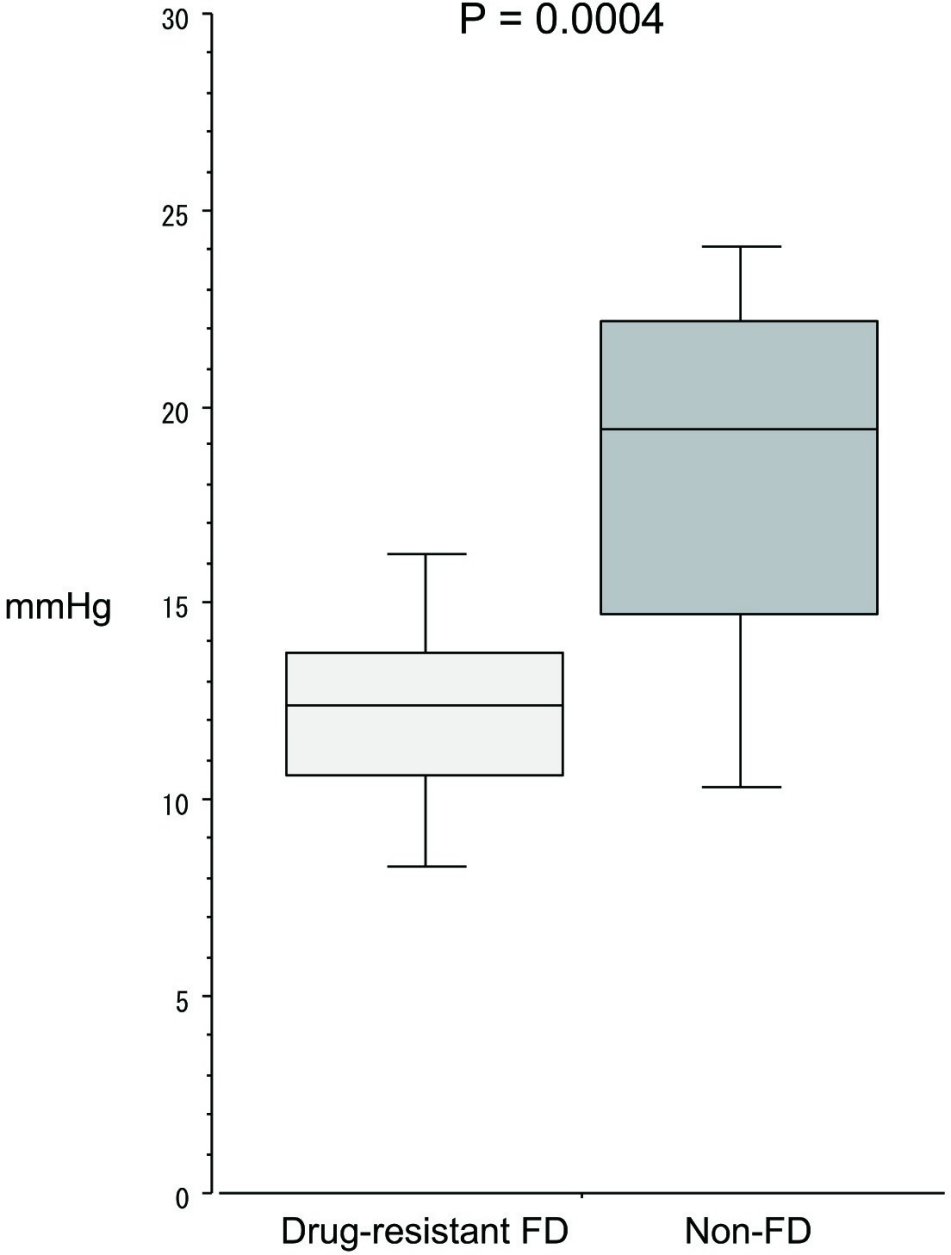
Gastric pressure at the time of bothersome symptom in the upper abdomen during a continuous CO_2_ injection.

**Fig 5:**
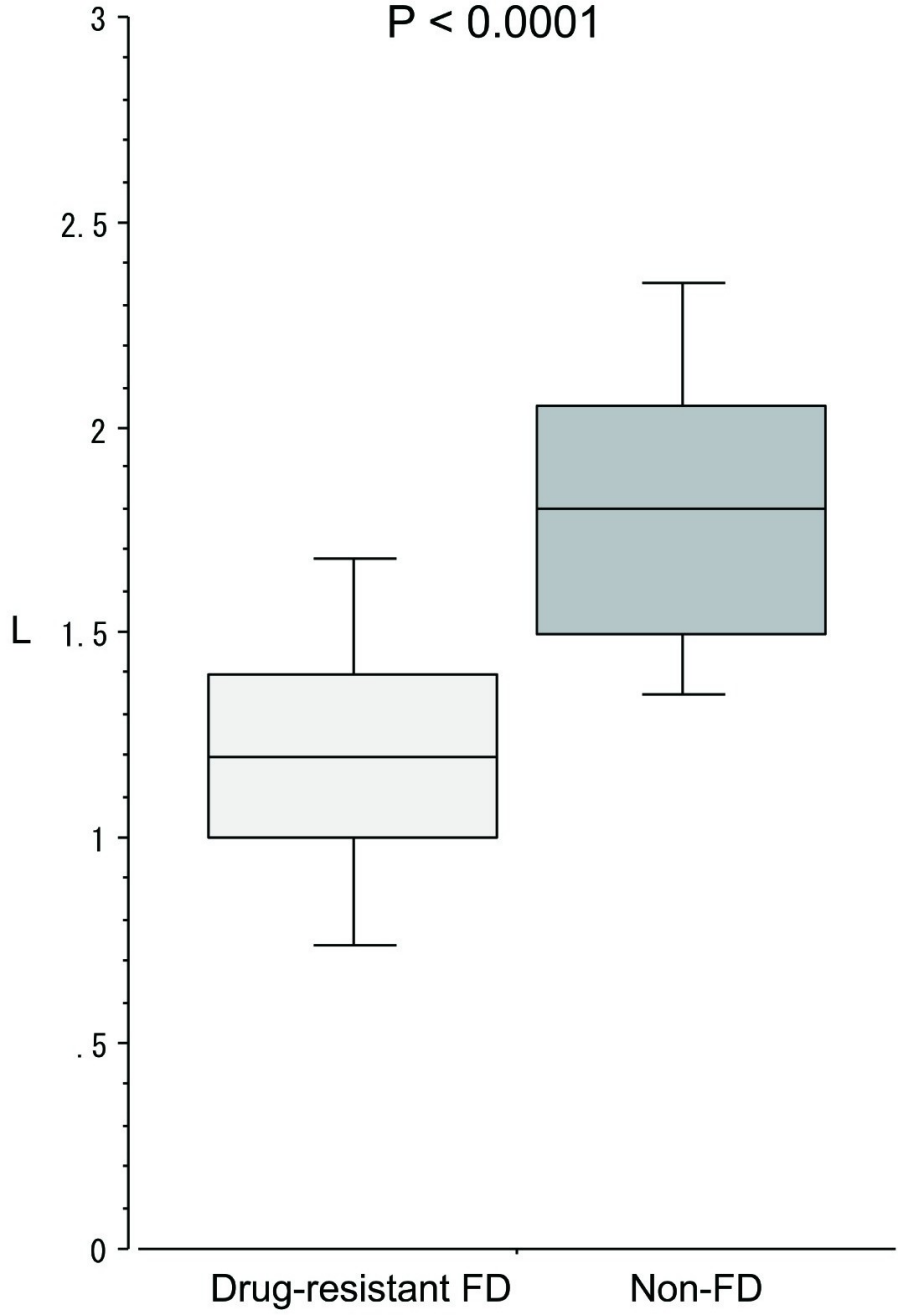
CO_2_ injection volume at the time of bothersome symptom in the upper abdomen during continuous CO_2_ injection.

Gastric pressure (Fig 4) at the time of awareness of bothersome symptoms was significantly (p = 0.0004) lower in the drug-resistant FD group (12.4 mmHg (10.7–13.8)) than in the non-FD group (19.5 (14.8–22.2)). Similarly, CO_2_ injection volume (Fig 5) was significantly (p < 0.0001) lower in the drug-resistant FD group (1.2L (1.0–1.4)) than in the non-FD group (1.8 (1.5–2.1)).

### Association between belching and bothersome symptoms

Frequency of belching before or at the time when bothersome symptoms occurred in the drug-resistant FD group was significantly (p = 0.0127) lower than in the non-FD group (Table 2).

**Table 2:** Presence of belching before or the same time of the onset of bothersome symptom after continuous CO2 injection under endoscopy in resistant FD group and non-FD group.

	Belching	
	+	-
Drug-resistant FD	5*	25
Non-FD	15	15

*P = 0.0127

Statistical analysis by Fisher’s exact test

## Discussion

Consistent with our previous findings [8], we demonstrated that the initial symptoms occurred with a lower gastric pressure and a smaller CO_2_ injection volume in the drug-resistant FD patients than in non-FD patients. Similarly, bothersome symptoms occurred with a lower gastric pressure and a smaller CO_2_ injection volume in the drug-resistant FD patients. This may reflect the impact of gastric mucosal hypersensitivity in drug-resistant FD patients.

We previously reported that the cut-off value of gastric pressure for drug resistance was 12.7 mmHg [8]. Based on this value, 28 of 30 patients in our study cohort had a gastric pressure of less than 12.7 mmHg. Moreover, all patients experienced symptoms with an injection volume of less than 1.25 L, which was the previously described cut-off value for CO_2_ volume [8]. This further emphasizes that gastric pressure and CO_2_ injection volume are suitable indicators of drug resistance and/or gastric hypersensitivity. are suitable indicators of drug resistance and/or gastric hypersensitivity. Although it is invasive, the barostat remains the gold standard test for assessing gastric hypersensitivity [12]. The endoscopic techniques described herein are easy to perform and more applicable to routine clinical practice.

Although a pressure sensor is required to measure gastric pressure, it has been reported that 30 mL of CO_2_ is injected per second using the endoscopic CO_2_ regulation unit (UCR). Indeed, the specific unit we used in the present study had an injection volume of 30–31 mL per second. As the approximate CO_2_ injection volume can be estimated based on the duration of injection, drug resistance and gastric hypersensitivity may be evaluated based on CO_2_ injection volume without the need for special equipment. Using our method, we plan to further examine the presence of drug resistance in FD patients prior to the start of their treatments.

In our previous study [8], we recruited GERD patients who did not have FD symptoms as the non-FD group. As we investigated the relationship between belching and bothersome symptom in this study, we excluded GERD patients from the non-FD group. Belching is common in GERD patients because the mechanism of belching is a transient lower esophageal sphincter relaxation [13, 14], which is also a mechanism of acid reflux [14, 15]. As such, we excluded GERD patients from our study subjects as GERD can lead to belching.

Our study revealed that patients in the drug-resistant FD group experience both the initial and bothersome symptoms at a lower gastric pressure and a smaller CO_2_ injection volume than those in the non-FD group. The primary aim of the study goal was not to describe the disease state of drug-responsive FD; rather, our aim was to examine the gastric pressure and CO_2_ injection volume at the time of awareness of initial and bothersome symptoms in drug-resistant FD. We observed that the symptoms improved in a small subset of patients with drug-resistant FD and their gastric pressure and CO_2_ injection volume were within the standard ranges. In future studies, we intend to examine a drug-responsive FD patient population to determine whether our findings are limited to drug-resistant FD.

The frequency of belching before or at the same time when bothersome symptoms occurred was significantly lower than that in drug-resistant FD group, suggesting that gastric pressure and gastric volume increase in the resistant group, compared with non-FD group because of less frequency of belching.

In the drug-resistant FD group, it was rare for the patients to experience belching before the onset of bothersome symptoms, as the threshold for bothersome symptoms would be lower than that for belching. In contrast, non-FD patients experienced belching before the onset of bothersome symptoms because the threshold for belching would be lower than that for the onset of bothersome symptoms. As a result, gastric pressure is less likely to be high and lead to bothersome symptoms in non-FD patients.

Acid suppression, acotiamide (prokinetic agent) and rikkunshito (Japanese herbal medicine) are recommended for the initial treatment of FD [10]. The second line treatment includes prokinetic agents except for acotimide and other Japanese herbal medicines except for rikkunshito [10]. However, patients in our study had persistent symptoms despite the use of these therapeutic agents. For these patients, clinical guidelines suggest psychosomatic treatment, including cognitive behavioural therapy as another line of treatment. However, it is not a common treatment option in Japan as the number of healthcare professionals that specialize in cognitive behavioural therapy is limited. We demonstrated that bothersome symptoms developed at a lower gastric pressure and a smaller CO_2_ injection volume in the drug-resistant FD group than in the non-FD group, suggesting that the symptoms were associated with gastric pressure and intragastric volume. In the treatment of FD, the basic treatment strategy is diet recommendation to reduce the intake volume. Also, it may be effective to reduce gastric pressure in addition to the diet recommendation. Acute administration of L-menthol is known to reduce gastric pressure in healthy subjects [16]. Although the administration of L-menthol reportedly does not affect the symptoms and gastric sensitivity [16], there are no studies that specifically examined its effects in drug-resistant FD patients. Future studies are needed to examine the effects of L-menthol administration in drug-resistant FD patients.

In our patient cohort, there were significant differences in the age, presence of gastric mucosal atrophy, and BMI. There is no consensus as to whether age is associated with FD, although several studies suggested that FD is more common in the younger population [17, 18]. Patients in the drug-resistant FD group were significantly younger than those in the non-FD group. Previously, a study examined the effect of age on the perception of gastric distention fullness measured by Barostat testing and demonstrated that the perception of fullness is reduced in an older population aged between 68 and 73 years compared with a younger population aged between 22 and 27 years [19]. While our patient population included younger patients in their 40s, we did not find a difference in perception between the younger and older populations. Furthermore, the age difference between the two populations in our cohort was 12 years, which is smaller than the previous study. As such, we assume that age did not affect our findings. Nonetheless, we intend to address the effect of age in future studies.

Gastric atrophy was more common in the non-FD group. This may be attributed to many of the patients in the non-FD group having ulcer scars, chronic gastritis or a history of *H*. *pylori* eradication. The degree of acid secretion in the two groups was considered to differ such that gastric acid secretion was higher in the drug-resistant FD group. Although gastric acid is involved in the symptoms of FD [20–22], patients in the drug-resistant FD group had symptoms that did not improve despite the use of PPI. This suggests that their symptoms were not a result of increased acid secretion. There is little evidence to reach a consensus regarding BMI [17, 23], however, it was significantly lower in the drug-resistant FD group in our patient cohort. Since patients in the FD group had drug-resistant bothersome symptoms, they did not eat much. Thus, their BMI was lower than non-FD group. There were no significant differences in other factors (sex and hiatal hernia) between the groups.

### Limitation

Our study was a single-center study with a limited number of patients. As such, there were differences in the age and proportion of patients with gastric mucosal atrophy between the groups. The diagnosis of our FD patients was based on the Japanese Gastroenterological Association criteria, not the Roma IV criteria [24].

In addition, our study population only consisted of patients with drug-resistant FD. In future studies, we intend to include patients with drug-responsive FD to determine whether our findings are characteristic of drug-resistant FD.

## Conclusions

Bothersome symptoms in drug-resistant FD patients develop at a lower gastric pressure and a smaller CO_2_ injection volume than in non-FD patients. They had also difficulties with belching, suggesting that this plays a role in the increase in gastric pressure and intragastric volume.
